# Sex Disparities in Efficacy in COVID-19 Vaccines: A Systematic Review and Meta-Analysis

**DOI:** 10.3390/vaccines9080825

**Published:** 2021-07-27

**Authors:** Alessia Bignucolo, Lucia Scarabel, Silvia Mezzalira, Jerry Polesel, Erika Cecchin, Giuseppe Toffoli

**Affiliations:** 1 Experimental and Clinical Pharmacology, Centro di Riferimento Oncologico di Aviano (CRO) IRCCS, Via Franco Gallini 2, 33081 Aviano, Italy; alessia.bignucolo@cro.it (A.B.); lucia.scarabel@cro.it (L.S.); silvia.mezzalira@cro.it (S.M.); ececchin@cro.it (E.C.); 2 Unit of Cancer Epidemiology, Centro di Riferimento Oncologico di Aviano (CRO) IRCCS, Via Franco Gallini 2, 33081 Aviano, Italy; polesel@cro.it

**Keywords:** SARS-CoV-2, COVID-19, vaccines, sex, gender, immune system

## Abstract

Sex differences in adaptive and innate immune responses have been shown to occur and anecdotal reports suggest that vaccine efficacy and safety may be sex-dependent. We investigated the influence of sex on the efficacy of COVID-19 vaccines through a systematic review and meta-analysis of clinical trials on COVID-19 vaccines. The safety profile of COVID-19 vaccines was also investigated. A systematic review included eligible articles published in three databases and three websites. A meta-analysis of available data, stratified by sex, was conducted. Statistical analysis was performed using the Hartung–Knapp–Sidik–Jonkman method, as well as influence and heterogeneity analysis. Pooled analysis showed significantly higher efficacy, measured as the rate of new COVID-19 cases, in men compared to women in the vaccine group (OR = 0.67, 95% CI 0.48–0.94). No sex differences were found in the rate of new cases in the control group (OR = 0.92, 95% CI 0.78–1.09). Safety profiles derived from pharmacovigilance reports appear to indicate increased toxicity in women. In conclusion, evidence of a potential role of sex in COVID-19 vaccine efficacy was described. It strengthens the need to include sex as a core variable in the clinical trial design of COVID-19 vaccines.

## 1. Introduction

As of 11 May 2021, there have been more than 158 million confirmed cases of COVID-19 with 3,299,764 deaths and a total of 1,206,243,409 vaccine doses administered.

SARS-CoV-2 infection indiscriminately affects the entire population, with incidence, severity and mortality of COVID -19 disease varying between sexes depending on several factors such as age, comorbidities and chronic diseases [1]. The exponential development of COVID-19 vaccines emphasizes the issue of their sex-related efficacy and safety. Indeed, sexual dimorphism has already been described in the context of innate and adaptive immune responses, expression of X-related genes, ACE2 receptor, sex hormone levels, and even microbiome composition [2,3,4]. Evidence on vaccines, including those for prevention of influenza, yellow fever, and hepatitis, seems to indicate higher immunoreactivity after vaccination in women compared to men [2]. Furthermore, adverse reactions appear to be more frequent and severe in women, who are also more likely to develop autoimmune and allergic reactions than men [5,6]. Currently, sex-disaggregated data for COVID-19 vaccines are still disjointed and incomplete and it is difficult to draw a comprehensive conclusion about the contribution of sex to vaccine efficacy and safety.

Sex-related factors, influencing both the subjects’ immunity and the vaccine outcomes, are still neglected in the clinical research, and clinical trials very rarely include a pre-planned sex-disaggregated analysis in their study design [4,7].

In the herein reported systematic review and meta-analysis, available data on the sex-specific efficacy, derived from clinical trials of COVID-19 vaccines, were investigated. The sex-specific safety profile of COVID-19 vaccines was also analyzed.

## 2. Methods

### 2.1. Systematic Review

This work used a systematic review methodology and adheres to the Preferred Reporting Items for Systematic Reviews and Meta-Analysis (PRISMA) guidelines [8]. The selected outcomes of the research were 1. efficacy, and 2. safety of COVID-19 vaccines in males as compared to females.

The systematic literature search was conducted on 30 April 2021 without language restriction using PubMed, the Cochrane Library, Scopus databases. Boolean operators AND/OR were used to combine search terms. Medical Subjects heading (MeSH) terms used for PubMed and Cochrane Library database included (“COVID-19” OR “SARS-CoV-2”) AND “Vaccines” and were filtered for “clinical trial” as article type, while in Scopus database the following string was used: “KEY ((“COVID-19” OR “SARS-CoV-2”) AND “Vaccine” AND “clinical trials”) AND (LIMIT-TO (DOCTYPE, “ar”)) AND (LIMIT-TO (EXACTKEYWORD, “COVID-19”) OR LIMIT-TO (EXACTKEYWORD, “Clinical Trial (topic)”) OR LIMIT-TO (EXACTKEYWORD, “SARS-CoV-2 Vaccine”) OR LIMIT-TO (EXACTKEYWORD, “SARS-CoV-2”)) AND (LIMIT-TO (SRCTYPE, “j”)). After duplicates were deleted, a systematic screening was conducted. Articles considered not pertinent (i.e., review, letters, expert opinions, case report, other vaccines, or COVID-19 pharmacological therapy) and pre-clinical studies were excluded. Simultaneous research was performed on the following websites: www.who.int (accessed on 30 April 2021), www.fda.gov (accessed on 30 April 2021) and www.ema.europa.eu (accessed on 30 April 2021) for additional records regarding COVID-19 vaccines. Particularly, we searched: in “COVID-19 vaccine” website area filtering by “press release/public statement” for Food and Drug Administration (FDA), in COVID-19 pandemic latest updates filtering by “vaccine” for European Medicines Agency (EMA) and in “Draft landscape and tracker of COVID-19 candidate vaccines” section for WHO. Vaccine and Related Biological Products Advisory Committee (VRBPAC) briefing documents and European Public Assessment Reports (EPAR) were assessed on FDA and EMA websites, respectively. Concerning WHO database, only the clinical trial section was taken into account and the study reports were screened. Only studies of COVID-19 vaccines reporting efficacy and/or safety sex-disaggregated data from the two above-mentioned strategies were included in the meta-analysis. This systematic research was performed independently by three authors (A.B., S.M. and L.S.).

### 2.2. Statistical Method

For each trial, the incidence of SARS-CoV-2 infection among people receiving either placebo or vaccine was calculated as the number of PCR-confirmed symptomatic COVID-19 patients divided by the number of enrolled subjects; the analyses were stratified by sex. Odds ratio (OR) for infection was calculated as the ratio between incidence rates. Considering the low number of studies, summary OR and corresponding 95% confidence intervals (CI) were calculated using random-effects models according to the Hartung–Knapp–Sidik–Jonkman method [9]. Statistical heterogeneity among studies was evaluated using the I^2^ and τ^2^ statistics [9]. Influence analysis was performed; pooled OR was calculated by omitting one study at a time. When at least two studies were available for both the cohort and the case–control design, summary relative risk (RR) was stratified by study design. The analyses were stratified by sex. The results of the meta-analysis were presented graphically, plotting the studies’ OR as a black square and the summary OR as a diamond. Statistical analyses were conducted using R 3.6 and statistical significance was claimed for *p* < 0.05 (two sided).

## 3. Results

### 3.1. Studies Selection

Running the search strategy on databases returned a total of 247 records. After removing the duplicates, 208 articles were identified and were then screened by titles, abstracts, and full text. 173 records were removed (167 not pertinent and 6 pre-clinical trials). The simultaneous search on the websites led to 283 records overall. By screening, 220 records were excluded whereas 63 (50 WHO records, 4 EPAR product information reports, and 3 VRBPAC-FDA Briefing Documents) were evaluated for eligibility.

**Figure 1:**
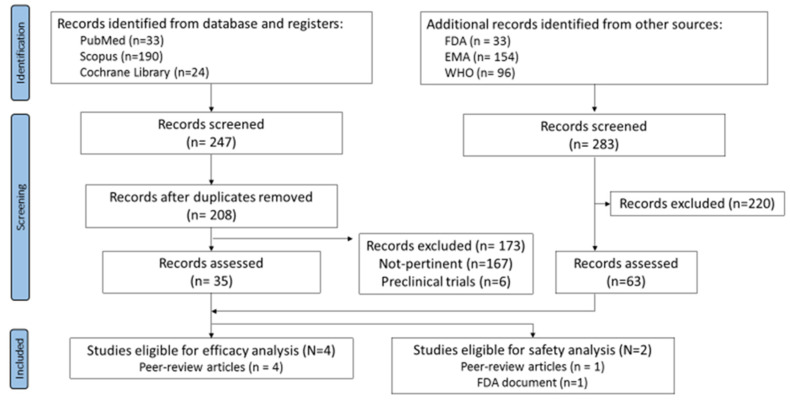
Flow-chart of systematic review.

Four peer-review articles reporting sex-disaggregated data for the efficacy outcome were identified. Only two studies met the inclusion criteria for the safety outcome; however, the data reported were different and not comparable, preventing the possibility to perform a meta-analysis on safety (Figure 1).

### 3.2. Summary of Efficacy

Efficacy data retrieved from the four studies highlighted by the systematic review are summarized in Table 1. Efficacy data were available for the mRNA vaccines, BNT162b2-BioNTech/Pfizer [10] and mRNA-1273-Moderna [11], and the non-replicative vector-based vaccines, Ad26.COV2.S-Johnson&Johnson/Janssen [12] and Gam-COVID-Vac-Gamaleja [13].

For all the vaccines considered, only symptomatic cases of COVID-19 confirmed by PCR test were included in the efficacy analyses. The number of symptomatic and PCR positive COVID-19 cases was evaluated at different time points according to the specific clinical protocol, type of symptoms considered, administration schedule, and type of vaccine.

For the BNT162b2 mRNA vaccine, developed by Pfizer/BioNTech, efficacy was evaluated as the onset of SARS-CoV-2 infection at least 7 days after the second dose in COVID-19 naive subjects before vaccination in 37,706 healthy volunteers: 19,075 males (50.6%) and 18,631 females (49.4%) [10]. The vaccine was demonstrated to be efficacious both in men (OR = 0.04, 95% CI 0.01–0.11) and in women (OR = 0.06, 95% CI 0.03–0.16).

The efficacy analysis within the phase III study of the mRNA-1273 vaccine, developed by Moderna, evaluated 30,351 volunteers: 15,985 males (52.7%) and 14,366 females (47.3%) [11]. The efficacy rate was measured as the first occurrence of symptomatic COVID-19 infection with an onset at least 14 days after the second dose administration in subjects that were seronegative at baseline [11]. Higher efficacy in the vaccine arm compared to placebo was reported for men (OR = 0.05, 95%, CI 0.02–0.13) and women (OR = 0.07, 95% CI 0.03–0.15). To date, Ad26.COV2.S-Johnson&Johnson/Janssen is the only adenoviral vector vaccine administered as a single intramuscular injection [14]. The efficacy, assessed as the onset of confirmed cases of COVID-19 infections occurring at least 14 and 28-days post-vaccination in seronegative population at baseline, was measured in 39,321 healthy subjects: 21,834 males (55.5%) and 17,479 females (44.5%) [14]. The 28-day post-vaccination results were considered for the analysis. Increased efficacy, compared to placebo, was observed either in males (OR = 0.30, 95% CI 0.22–0.41) and females (OR = 0.40, 95%, CI 0.29–0.54). Gam-COVID-Vac, developed by Gamaleja, enrolled for the efficacy analysis 19,866 healthy volunteers: 12,158 males (61.2%) and 7708 females (38.8%) [13]. The efficacy outcome was evaluated as COVID-19 confirmed cases at least 21 days after the first dose [13]. The vaccine was effective both in men (OR = 0.06, 95%, CI 0.03–0.13) and in women (OR = 0.13, 95%, CI 0.06–0.27).

### 3.3. Meta-Analysis

**Figure 2:**
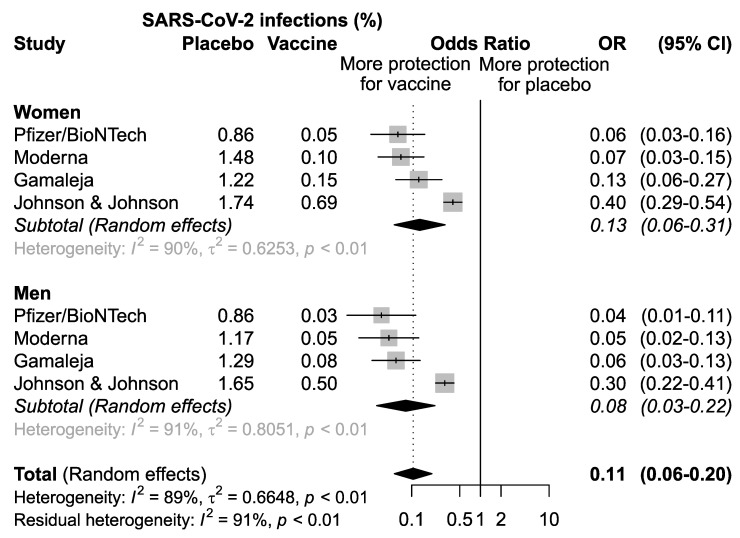
Odds ratio (OR) and corresponding 95% confidence intervals (CI) for new infection of SARS-CoV-2 in healthy subjects receiving vaccine versus placebo.

A first meta-analysis of the pooled studies was performed to assess the efficacy of vaccination in men and women separately (Figure 2). The results confirmed that overall, the four vaccines analyzed are effective both in women (OR = 0.13, 95% CI: 0.06–0.31; *p* < 0.01) and in men (OR = 0.08, 95%CI 0.03–0.22), *p* < 0.01) (Figure 2).

**Figure 3:**
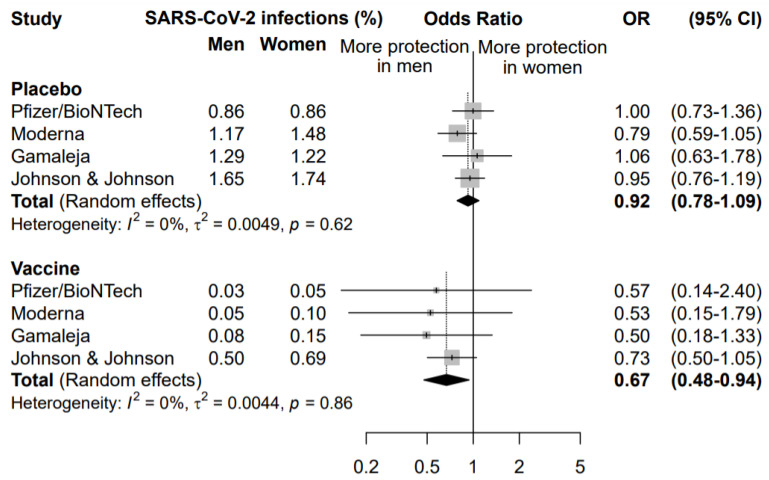
Odds ratio (OR) and corresponding 95% confidence intervals (CI) for new infection of SARS-CoV-2 in men versus women in subjects receiving placebo or vaccine.

A second meta-analysis was performed to compare the effect of the COVID-19 vaccination in men and women (Figure 3).

The rate of symptomatic infected individuals in men was compared to the rate of infection in women, separately for the placebo and the vaccine arm. This analysis highlighted that vaccination was more effective in preventing COVID-19 disease in men than women (OR = 0.67, 95% CI 0.48–0.94). The same analysis in the placebo arm did not highlight any significant difference between males and females in the rate of new symptomatic COVID-19 cases (OR = 0.92, 95% CI 0.78–1.09), supporting a specific sex effect on the vaccination efficacy.

The sensitivity analysis among subjects enrolled in the vaccine arm, conducted excluding one study at a time, did not highlight any influent study, with an OR ranging between 0.52 excluding Johnson&Johnson/Janssen and 0.70 excluding Gamaleja (Figure 3). Nonetheless, it is worth noting that the study by Johnson&Johnson/Janssen reported the highest incidence of new COVID-19 cases in the vaccine arm in both men and women, with a narrower confidence interval. This also impacted the OR of comparison between men and women that resulted 0.73 versus the other three studies ranging between 0.50 and 0.57. This could be related to a lower overall efficacy of the vaccine as compared to the others and to different study designs and criteria for evaluating symptomatic COVID-19 cases.

### 3.4. Summary of the Safety

For the evaluation of the safety outcome, data from mRNA-1273-Moderna [11] and Ad26.COV2.S-Johnson&Johnson/Janssen [12] were available. However, toxicity endpoints reported in the two studies were heterogeneous, thus preventing the possibility to perform a meta-analysis of the data; mRNA-1273-Moderna [11] data focus on any non-fatal serious adverse event (nf-SAE), whereas Ad26.COV2.S-Johnson&Johnson/Janssen [12] report describes only the occurrence of venous thromboembolic events.

A total of 30,350 subjects treated with mRNA-1273-Moderna vaccine were evaluated for safety: 14,355 (47.3%) females and 15,995 (52.7%) males. In the vaccine arm, a total of 7 (4.8%) cases of nf-SAEs were described: 6 (85.7%) in females, and 1 (14.3%) in males. Five events (3.3%) were reported in the placebo group: 1 (20%) in a female and 4 (80%) in males [15]. These adverse effects included intractable nausea/vomiting, facial swelling, rheumatoid arthritis, dyspnea with exertion, peripheral edema, autonomic dysfunction, B-cell lymphocytic lymphoma, polymyalgia rheumatic, paresthesia, anxiety, procedural hemorrhage, pulmonary embolism and pneumonia, and myocardial infarction. A causality assessment analysis highlighted only intractable nausea/vomiting and facial swelling as likely related to the vaccine administration. These events were reported only in women enrolled in the vaccine arm. All of them, however, presented an underlying condition that could have triggered the adverse event occurrence (i.e., one case of intractable nausea/vomiting with a previous history of headaches and severe nausea requiring hospitalization, two cases of women with facial swelling with a previous history of dermal filler cosmetic injections).

A total of 6736 healthy subjects were included in the safety analysis of Ad26.COV2.S-Johnson&Johnson/Janssen vaccine: 3252 (48.3%) females and 3484 (51.7%) males. Thrombotic events included deep vein thrombosis, pulmonary embolism, transverse sinus thrombosis/cerebral hemorrhage. Specifically, 10/11 (90.9%) cases of such adverse reactions were reported in the vaccine group for males and 1/11 (9.1%) for females, while 3/3 (100%) cases in the placebo arm all occurred in males [12]. Only 1 case (male) of deep venous thrombosis was considered related to vaccine administration.

## 4. Discussion

The influence of sex on vaccine efficacy remains poorly understood, although it is widely recognized as a noteworthy topic. Sex-related differences in vaccine efficacy and safety, based on immunological, genetic, and hormonal backgrounds, have been previously reported emphasizing a possible influence of sex even on the outcome of COVID-19 vaccines [16,17,18].

To the best of our knowledge, this meta-analysis is the first to report pooled sex-disaggregated data from COVID-19 vaccines clinical trials focusing on a possible role of sex on their efficacy, assessed as risk reduction of a new infection.

The meta-analysis includes available sex-disaggregated data from BNT162b2-BioNTech/Pfizer, mRNA-1273-Moderna, Ad26.COV2.S-Johnson&Johnson/Janssen, which have already been approved by the FDA and EMA, and Gam-COVID-Vac-Gamaleja, which is still under review by the EMA.

The pooled data showed a significantly increased efficacy in men compared to women in the vaccine group. Following vaccination, males appear to have a 33% reduction in the overall risk of developing COVID -19 compared to females. This highlights the trend towards increased efficacy observed in each of the four clinical trials when analyzed separately. No sex differences in the development of COVID-19 disease were observed in the control arms.

Some limitations could be derived from the meta-analysis. In particular, the studies considered exhibited some differences that could represent bias: (1) type of COVID-19 vaccines; (2) administration schedules; (3) timing of efficacy endpoint. However, the novelty of the subject and the compelling need to obtain more detailed information on the COVID-19 vaccines and all the factors potentially affecting their outcome should deserve attention. In addition, such observations are not consistent with previous anecdotical reports of other vaccines (for instance measles, tuberculosis, hepatitis, yellow fever) that have been shown to be more effective in women than in men [3,19].

The safety analysis included only two studies, an FDA document on mRNA-1273-Moderna [15] and a published clinical trial on Ad26.COV2.S-Johnson&Johnson/Janssen [12]. The Moderna study indicated that nf-SAEs were more common in women [15]. No data about overall nf-SAE were reported by Johnson&Johnson/Janssen study. The latter indicated that deep venous thromboembolic events seem to occur more frequently in men. However, this observation should be viewed with extreme caution as most thromboembolic events occurred in men in the placebo arm as well. These data are consistent with a sex-related imbalance of deep venous thrombosis condition, which is also more common in men in the general population [20,21].

One of the innovations resulting from the COVID-19 pandemic is the wider use and also regulatory acceptance of real-world data and pharmacovigilance reports [22]. On 8 May 2021, EUDRAvigilance reported sex-disaggregated data on the safety of approved COVID-19 vaccines. For the four vaccines analyzed (BNT162b2-BioNTech/Pfizer, mRNA-1273-Moderna, Ad26.COV2.S-Johnson&Johnson/Janssen and ChAdOx1nCOV-19-Oxford/AstraZeneca) suspected adverse drug reactions ranged from 70.9% to 76.0% in women and from 22.4% to 28.6% in men [23], suggesting an increased incidence of toxicity in women compared with men. However, it must be considered that women have a greater tendency to report adverse drug reactions [24,25].

Very rare cases of thrombotic events (Cerebral Venous Sinus Thrombosis) with thrombocytopenia have been reported by pharmacovigilance agencies following ChAdOx1nCOV-19-Oxford/AstraZeneca and Ad26.COV2.S-Johnson&Johnson/Janssen vaccination. Those adverse reactions occurred predominantly in women for both vaccines [26,27]. In both studies, female subjects were younger than 60 years suggesting sex and age-related effects.

Currently, only sparse and incomplete data are available from the registration trials of COVID-19 vaccines and sex-disaggregated data are difficult to retrieve. This leaves many questions unanswered, such as sex-specific knowledge on the duration of vaccine protection, on the neutralizing antibodies persistence, on the differential efficacy of the vaccination on SARS-CoV-2 genetic variants, and the effect of pregnancy on vaccine response. This reinforces the need to include sex as a primary objective in clinical trials.

In conclusion, preliminary data available on COVID-19 vaccines indicate potential sex-related differences in efficacy and safety. In the pandemic era, the correlation between sex and COVID-19 vaccine efficacy and safety should be considered in enhancing vaccine decision-making programs. Indeed, these results could be useful in the clinical setting for potential adjustments to vaccination schedules or required booster vaccinations. Further investigations are needed to design specific clinical trials that consider sex as a key variable in the study endpoints.

## Figures and Tables

**Table 1:** Available vaccines with sex-disaggregated data for efficacy.

Sponsor	Approval Date	NCT	Phase	Study Design	Schedule (d)	Population N (%)	Efficacy			Ref
							Placebo-Control n/N (%)	Vaccine n/N (%)	% (95% CI)	
Pfizer/BioNTech	FDA 11/12/2020	NCT04368728	II/III	RCT	2 IM doses	All: 37706	All: 162/18846 (0.86)	All: 8/18860 (0.04)	All: 95.0 (90.0–97.9)	[10]
	EMA 21/12/2020			1:1	(0, 21)	M: 19,075 (50.6)	M: 81/9436 (0.86)	M: 3/9639 (0.03)	M: 96.4% (88.9–99.3)	
				blinded		F: 18,631 (49.4)	F: 81/9410 (0.86)	F: 5/9221 (0.05)	F: 93.7% (84.7–98.0)	
Moderna	FDA 18/12/2020	NCT04470427	III	RCT	2 IM doses	All: 30351	All: 185/14073 (1.31)	All: 11/14134 (0.1)	All: 94.1% (89.3–96.8)	[11]
	EMA 06/01/2021			1:1	(0, 28)	M: 15,985 (52.7)	M: 87/7462 (1.17)	M: 4/7366 (0.1)	M: 95.4% (87.4–98.3)	
				blinded		F: 14,366 (47.3)	F: 98/6611 (1.48)	F: 7/6768 (0.1)	F: 93.1% (85.2–96.8)	
Gamaleja	Russia 11/08/2020	NCT04530396	III	RCT	2 IM doses	All: 19866	All: 62/4902 (1.26)	All: 16/14964 (0.1)	All: 91.6% (85.6–95.2)	[13]
	Rolling review EMA 04/03/2021			3:1	(0, 21)	M: 12,158 (61.2)	M: 39/3015 (1.29)	M: 7/9143 (0.1)	M: 94.2% (87.2–97.4)	
				double-blinded		F: 7708 (38.8)	F: 23/1887 (1.22)	F: 9/5821 (0.2)	F: 87.5% (73.4–94.2)	
Johnson & Johnson (Janssen)	FDA 27/02/2021	NCT04505722	III	RCT	1 IM dose	All: 39321	All: 193/19178 ^§^ (1.01)	All: 66/19306 ^§^ (0.3)	All: 66.1% (55.0–74.8)	[12]
	EMA 11/03/2021			1:1		M: 21,834 (55.5)	M: 176/10649 (1.65)	M: 54/10764 (0.5)	M: 69.8% (58.9–78.2)	
				double-blinded		F: 17,479 (44.5)	F: 148/8525 (1.74)	F: 59/8538 (0.7)	F: 60.3% (46.0–71.2)	

^§^ Efficacy data regarding the Johnson&Johnson/Janssen vaccine were reported as Per-Protocol Set with the first occurrence of moderate to severe/critical COVID-19 with onset at least 28 days after vaccination (sex-disaggregated efficacy data included non-centrally confirmed cases, while efficacy for all population included only centrally confirmed cases). Abbreviations. n: cases; N: total subjects; M: male; F: female; RCT: randomized clinical trial.

## Data Availability

The data presented in this study are available in the article.
